# Doctors have an ethical obligation to ask patients about food insecurity: what is stopping us?

**DOI:** 10.1136/medethics-2021-107409

**Published:** 2021-07-14

**Authors:** Jessica Kate Knight, Zoe Fritz

**Affiliations:** 1 Department of Acute Medicine, School of Clinical Medicine, University of Cambridge, Cambridge, UK; 2 Department of Acute Medicine, The Healthcare Improvement Studies Institute, Cambridge, UK; 3 Cambridge University Hospitals NHS Foundation Trust, Cambridge, UK

**Keywords:** ethics, education for health care professionals, health promotion, social aspects

## Abstract

Inadequate diet is the leading risk factor for morbidity and mortality worldwide. However, approaches to identifying inadequate diets in clinical practice remain inconsistent, and dietary interventions (on both individual and public health policy levels) frequently focus on facilitating ‘healthy choices’, with limited emphasis on structural constraints. We examine the ethical implications of introducing a routine question in the medical history about ability to access food. Not collecting data on food security means that clinicians are unable to identify people who may benefit from support on an individual level, unable to consider relevant dietary risk factors for disease and disease progression and unable to monitor population trends and inequalities in dietary access in order to design effective policy interventions. We argue that the current lack of routine screening for food insecurity is inconsistent with our approach to other health behaviours (eg, smoking and alcohol use), as well as with doctors’ frequent informal role as gatekeepers to the food aid system, and recent calls for governmental action on food insecurity and health inequalities from individual clinicians and professional bodies. Potential ethical barriers to asking patients about food security are addressed, including concerns about stigma, limiting autonomy, fair resource allocation, unclear professional remits and clinicians’ ability to offer effective interventions. We suggest that there is an ethical imperative for doctors to ask patients about their ability to access healthy food. Gathering this data provides a valuable first step in re-framing the social determinants of health as modifiable risks, rather than inevitable inequities.

## Introduction

No one disputes the relationship between diet and health: our bodies reflect what we eat, and—just as importantly—what we do not or cannot eat. Inadequate diet is the leading risk factor for morbidity and mortality globally, responsible for 11 million deaths in 2017, and surpassing the effects of other behavioural risk factors including tobacco use, alcohol consumption, recreational drug use and unsafe sex combined.^1^ Improvements in diet have the potential to prevent one in five deaths worldwide.^1^ In the UK, patterns are similar, with 10.8% of DALYs (Disability-adjusted Life Years) attributable to suboptimal diet.^2^ And yet asking about diet is not routinely part of the medical history, unlike questions about other determinants of health such as smoking and alcohol. Here, we first examine the interrelationship between diet, health and healthcare delivery; then explore whether there is an ethical obligation to ask about food insecurity, and finally address some of the ethical counterarguments to doing so.

### Diet and health

The health effects of dietary risks are mediated by multiple intersecting causal pathways, including those associated with ‘overnutrition’ and atherogenic diets, as well as those related to underconsumption of key micronutrients and macronutrients. Although a large proportion of policy and public attention is paid to the effects of excess sugar, salt and saturated fat, the leading dietary risk factors for mortality (other than a high-sodium diet) are diets low in whole grains, fruit, nuts and seeds, vegetables and omega-3 fatty acids.^1^ Crucially, harmful underconsumption and overconsumption can co-exist, resulting in the so-called ‘double burden of malnutrition’, where populations are simultaneously affected by micronutrient deficiencies, underweight and childhood stunting as well as overweight, obesity, and related non-communicable diseases.^3^

### Hunger, malnutrition and ‘food insecurity’

‘Food insecurity’, or ‘food poverty’, has been defined as the inability to consume an adequate quality or quantity of food in socially acceptable ways or the uncertainty that one will be able to do so.^4^ The term has been criticised as simultaneously too specific, in narrowly focusing on food without acknowledging the other, broader implications and causes of poverty; and not specific enough, in avoiding the reality that an ‘insecure’ ability to access food frequently simply leads to hunger.^5 6^ However, the concept of ‘food insecurity’ remains useful, in that it captures the existence of external dimensions (including poverty, isolation, and mental or physical ill-health), which constrain access to a good diet. Food insecurity is distinct from (though may contribute to) malnutrition, which may be related to disease as well as inadequate oral intake, and is frequently treated by medical professionals.

Food insecurity in the UK has been a focus of growing public attention over the past decade, particularly with the rise in the numbers and visibility of ‘food banks’, third-sector organisations providing food to those in acute need. The Trussell Trust network, which accounts for around 60% of UK food banks, report a tripling of their food parcel provision in the decade since 2010, and numbers of independent food banks (unaffiliated with the Trussell Trust) have increased correspondingly.^5 7^ Further increases in food bank use have been reported as a result of the coronavirus pandemic, accompanied by a series of calls, led by Marcus Rashford, for governmental action on child food poverty and ‘holiday hunger’.^8^

The relationship between food insecurity and poor health is mediated through multiple mechanisms, including constrained dietary options (due to cost and the food bank ‘surplus’ supply model), compensatory strategies (such as skipping meals or relying on energy-dense foods), inability to control diet in chronic disease (eg, worse diabetes control) and the chronic stress of not knowing whether there will be enough to eat.^5 9–13^ In 2014, the Faculty of Public Health called on the government to take action on food insecurity by reversing the rising costs of healthy foods and falls in wages and welfare payments.^14^ More recently, the RCPCH and the BMA issued public statements in 2020 calling for half-term provision of free school meals on health grounds.^15 16^

### Doctors and (non-dietary) health behaviours

A classic dilemma in public health ethics concerns the roles of the doctor and the state in changing health behaviours and the extent to which it is acceptable to restrict the autonomy of an individual or population for the sake of their own or others’ health.

Broadly, existing interventions can be divided into those supporting an individual to change their behaviour (eg, smoking cessation services, weight loss programmes) and those altering the behaviour of a population via ‘nudges’ or financial incentives/disincentives (eg, taxation on tobacco, alcohol or sugar).^17^ In the latter case, the potentially coercive or paternalistic policy is frequently justified by its overall positive impact on health and health equity, as ‘unhealthy’ behaviours are more common in people living on low incomes.^18^

Certain health behaviours are routinely screened for in both primary and secondary care. Medical students are taught to enquire about patients’ smoking status and use of alcohol and recreational drugs as part of the ‘social history’.^19^ Justifications for this include the relevance of behaviours to diagnostic reasoning, as well as the ability to offer appropriate preventative healthcare alongside acute treatment. All NHS-funded providers are mandated in the NHS Standard Contract to screen for smoking and alcohol consumption, provide brief advice and offer referral to specialised services. This is justified because it may ‘reduce the burden on the NHS, premature mortality and morbidity (and) health inequalities’.^20^ Implementation of the ‘Ottawa Model’ for smoking cessation, in which smoking status is identified and documented for all inpatients, and intervention and follow-up are provided, is known to significantly increase cessation rates 6 months post-discharge.^21^

### Doctors and dietary health behaviours

There is no specific UK guidance recommending that clinicians routinely ask patients about their dietary habits or about barriers to accessing a ‘healthy’ diet; NICE’s recommendations are based on encouraging a balanced diet and screening for malnutrition.^22 23^ In the USA, where food insecurity has been annually monitored through the National Food Security Survey since 1990, there have been calls to integrate routine screening of diet and food insecurity into clinical practice. The American Heart Association argue that health impacts of poor diet and the potential for healthcare cost reduction provide a strong rationale for the implementation of a universal dietary screening tool in the electronic health record, while both the American Academy of Paediatrics and the American Association of Family Physicians recommend universal screening with the Hunger Vital Sign, a two-question tool to identify food insecurity.^24–26^

## Is there an ethical obligation to ask about food insecurity?

### Biological and ethical rationale for asking during initial diagnosis

A primary purpose of the medical history is for the clinician to gather relevant information required to construct a differential diagnosis and create a shared plan for care. Diet broadly, and food insecurity in particular, are of sufficient relevance to disease (equivalent to smoking and alcohol histories) to merit routine inclusion in the social history, particularly since malnutrition is both common among people presenting to acute services and has a significant influence on morbidity, mortality and recovery of functional status.^27^ Briefly, routine enquiries about access to a healthy diet could then be followed up, where appropriate, with a more detailed history based on the clinical picture.

Some specific diagnoses that could be ascertained through dietary history include the following:

1. Micronutrient deficiencies, electrolyte imbalances or refeeding syndrome—this can be caused by a restricted diet, due to physical, financial or psychological inability to access varied foods, or to voluntary restriction (eg, veganism).^28^
2. Protein–calorie malnutrition—this can be caused by inadequate oral intake, particularly in older people with low incomes, in addition to more commonly treated ‘biological’ causes such as malabsorption or cachexia.^29^
3. Type 2 diabetes mellitus—food insecurity is independently associated with an increased risk of developing type 2 diabetes.^30^
4. Poor medication adherence or efficacy as an explanation for ongoing symptoms—food insecurity has been linked to reduced adherence to medications that must be taken at specific times, reduced maintenance of therapeutic drug levels and worse disease control, for example, in HIV antiretroviral therapy.^31^

In addition to providing information relevant to diagnosis and acute management, a person’s social situation frequently informs safe discharge planning in inpatient settings and referrals to social care by GPs. Doctors are comfortable routinely asking about this (marital history, help at home, etc). Ability to access food is ethically equivalent in terms of balancing perceived infringement of privacy (‘why do you want to know what I have in my fridge? What does it matter to you who I live with?’) with achieving good health outcomes: an empty fridge has shown to be a significant predictor of early readmission among older adults.^32^

A dietary history is relevant across the full range of clinical environments: in secondary care, doctors’ awareness of dietary challenges may inform their initial diagnosis, referral for dietetic support within hospital and plans for discharge. Meanwhile in primary care, clinicians may better placed to offer brief advice, referral or signposting to local support groups and services on a longer-term basis, integrated with a biopsychosocial model of medical care. In the face of clear evidence concerning the impact of food insecurity on health outcomes, it is ethically inconsistent to avoid discussing factors affecting access to a good diet in clinical settings.

### Integrating dietary support with management of long-term conditions

Introduction of routine screening for food insecurity has the potential to allow patients and clinicians to create more effective shared plans for dietary management of long-term conditions and to minimise shame felt by some people experiencing food insecurity when given inappropriate ‘lifestyle advice’. These feelings appear to be prevalent: participants in a study in north east Scotland believed that their GP was unaware of their struggle to afford food and expressed reluctance to spontaneously confide in healthcare professionals, due to concerns over wasting clinicians’ time, embarrassing them or their inability to help.^12^ A related study of healthcare professionals found mixed awareness of the issue, though some practitioners specified occasions when their patients’ illnesses had been specifically worsened by their food insecurity (eg, inability to maintain a high-calorie diet in COPD (Chronic Obstructive Pulmonary Disease), or a low-carbohydrate diet in diabetes.)^11^

Long-term conditions are common in those with food insecurity: nearly 75% of people who have used a Trussell Trust food bank have at least one such disease,^5^ and evidence suggests that experience of food insecurity undermines people’s ability to manage their long-term conditions,^5 12 33 34^ including skipping meals and cutting back on medication.^12^ Those living with both diabetes and food insecurity, for example, have worse glycaemic control than those without food insecurity; 30 the control is improved on receipt of adequate aid.^35^

Discussion of food insecurity need not be confined to conversations about a modifiable risk after risk-related conditions have arisen: primary as well as secondary prevention should be encouraged. Routine dietary screening, particularly in primary care, provides the option of offering support and signposting to anybody at risk of experiencing food insecurity and interrupting the cyclical relationship between poor dietary access and development of disease.^24^

### Designing effective support systems

Health and social care professionals, including doctors, currently act as gatekeepers to the UK’s rapidly growing food aid system^34^—over 60% of independent food banks require referrals from a third party.^7^ Despite this, food banks are commonly funded entirely by charitable grants and public donations and run by volunteer labour.^33 34^ There is also interprofessional variability in knowledge of local food aid services and frequency of referral.^11^ Given the inconsistent and informal organisation of current systems, doctors’ and patients’ frequent sense of helplessness in the face of food insecurity is perhaps unsurprising.

Significant gaps also remain in current approaches to measuring the prevalence of food insecurity on local and regional levels in the UK and in the ability of existing data to link experience of food insecurity to specific health outcomes.^36 37^ Monitoring in healthcare settings has the potential to meet this unmet need, informing epidemiological research as well as local authority or CCG funding and policy decisions; for example, by explicit inclusion of food insecurity data into Health (or Health Equity) Impact Assessments.

The current informal referral ‘system’ risks both missing opportunities to provide effective support and passively institutionalising food banks as a permanent part of the UK welfare support infrastructure. Explicitly acknowledging the extent of reliance of healthcare providers and other statutory services on charitable food aid organisations would instead promote working in partnership to design evidence-based improvements in support services—for example, many food bank providers and anti-poverty campaigners push for a ‘Cash First’ approach, ensuring people receive adequate financial assistance rather than emergency food.^38^ Data collected in healthcare settings may be an influential tool in displaying the efficacy or otherwise of current systems and in advocating for change when needed.

### Improving clinical practice

Doctors have a primary duty to improve clinical practice and to ensure that their care is reflective of both progress in biomedical research and the changing needs of the people they serve. Although food insecurity in the UK is not a new problem, the high profile of the issue in recent months provides a crucial opportunity to make changes which ensure that healthcare services adequately meet the needs of food-insecure patients and reflect the clear consensus that there can be no place for hunger within a just society.

There are frequent public calls for governmental action on food insecurity by medical professional bodies,^15 16^ but as well as this vital broader policy change, it is important that these are also accompanied by change within healthcare services. The medical history provides a powerful tool for shaping individual attitudes and institutional cultures: Moscrop *et al* 39 argue that by remaining effectually ‘blind’ to social determinants of health (even those, like food insecurity, which are relatively downstream), ‘doctors help to conceal these problems from public view and from the political agenda… Ending the complicity of the medical profession in health and healthcare inequities begins with data gathering’*.”*

Routinely recording people’s ability to access the food they need, rather than simply providing advice on ‘healthy choices’, provides one small step to creating a healthcare system which truly promotes equal access to health for all.

## Addressing the counterarguments: potential ethical barriers to asking about food insecurity

### Stigma and trust

One prominent concern about introducing questions about food security into healthcare settings is the potential of damaging the therapeutic relationship by eliciting shame and perpetuating self-blaming stigmas associated with being unable to reliably access food. Poverty itself may be experienced as shameful,^40^ and food aid is frequently positioned as an act of ‘charity’ rather than fulfilment of a basic right, invoking an idea of ‘compulsory gratitude’ and a lack of self-determination, which can lead to humiliation.^41^

However, advocates of a ‘public health approach’ to issues such as knife crime or substance use contend that treating something as a health concern, not an individual failing, can help to promote support rather than stigmatisation.^42^ Though not directly analogous, framing access to food in terms of health and the right to a good diet, rather than relegating responses to ‘charity and chance’, may have a similar effect.^4^

Healthcare professionals are used to discussing difficult issues: pain, dying, continence, sexual problems and psychological trauma are part of everyday medical and nursing practice. Future clinicians receive communication skills training allowing them to discuss these issues with sensitivity, empathy and an attention to power imbalances in therapeutic relationships. There is no reason why it should be impossible to create the necessary training to enable food security and income to be discussed with equivalent care and dignity, minimising the provocation of shame.

### Respecting autonomy

Asking people about their ability to access food or signposting to sources of support with food or finances may potentially be seen as an intrusion into a person’s freedom to direct their own life.

Similar concerns may be raised about many measures to address ‘lifestyle diseases’ in medical practice. Advice about healthy habits and ‘disincentive’ policies to change population behaviour, such as the sugar tax, inevitably restrict individual liberty and may be seen as paternalistic. It has been argued that if lifestyle-induced ill health is due to a poverty of options, it is counterintuitive to further restrict limited options with ‘disincentive’ policies, which inevitably have the greatest impact on people with the least (economic) ability to choose.^18^

There are, however, existing intrinsic constraints on people’s ability to choose at food banks, and no unified ethical basis for good practice which ensures respect for autonomy in food aid services, though frameworks based on the ‘social empathy’ model and ‘capability approach’ have been proposed.^43^ ‘Means paternalists’ argue that we should accept people’s goals and aim at steering (or nudging) people’s behaviour towards those goals^17^—and in doing so increase their long-term ability to make autonomous choices. Formalising existing informal systems of referral from healthcare professionals (who have an explicit duty to protect autonomy) into food aid services may therefore act to promote, rather than limit, freedom of choice.

### Justice and resource allocation

Introduction of questions about food security into routine practice involves a use of healthcare resources, both in terms of the already stretched time of individual clinicians and the financial implications of any further programmes implemented as a result of data gathering. It is therefore reasonable to question whether this would be a just use of limited resources available within the healthcare system. Fritz and Cox^44^ propose a framework with which to ensure that conflicting needs are considered fairly using an application of Rawlsian principles including equity of access, openness, just savings and the difference principle, to ensure that justice is embedded in the healthcare system.

These principles may be applied to demonstrate that further allocation of healthcare resources towards routinely asking about diet and improving dietary access would be a just use:

- The difference principle states that primary goods should be distributed equally unless an unequal distribution would make the least advantaged in society materially better off than they would be under strict equality.^45^ The (physical and financial) ability to follow dietary advice is currently distributed unequally, restricting the ability of many to act as ‘normal and fully cooperating members of society over a complete life’.^46^ Physicians’ time spent in routinely eliciting a dietary history—which would take longer for some patients than others—would be justified in increasing the opportunities for those who were least advantaged, both through individual support, and through the data generated to guide policy and interventions.
- The just savings principle promotes intergenerational justice: investment in future health via preventative healthcare (such as promoting equal dietary access for all) is reasonable despite a potential opportunity cost at the present.
- Equity of access is enshrined in the NHS Constitution, yet there is an emphasis on funding interventions which aim to change individual dietary behaviours in spite of evidence that dietary access is also constrained; the role of the NHS in ensuring or at least facilitating access to nutritious food deserves further exploration.
- Openness means that decision-making should be transparent, and the ethical basis for allocation of resources accountable to the public. The normative basis for current allocation of healthcare funds to dietary health promotion initiatives must therefore be explicit.

The philosophical justification for improving knowledge about food access, as well as access to food, is therefore robust. Whether this falls into the remit of healthcare professionals is another question hat needs addressing.

### Professional and policy remits

Further integration of food aid into healthcare could be argued to shift institutional responsibility for hunger to healthcare professionals and distract from the responsibilities of politicians and welfare and economic policy reform. However, healthcare professionals (especially GPs) already give advice about diet, gatekeep entry into the food aid system and treat the eventual consequences of diet-induced or diet-exacerbated disease. Explicitly integrating assessment of food insecurity into healthcare, and documenting the extent of this reliance on voluntary organisations, may act to further incentivise development of effective policy responses to upstream causes such as income inequality, and help monitor health and social care costs (or benefits) of policy changes.

### Ability to intervene

It is evident that existing interventions to address food insecurity are not always working,^47–49^ so it is reasonable to be concerned about the ethical implications of ‘screening’ for a condition that does not currently have an effective ‘cure’. Doctors may feel impotent to deal with food insecurity, even if they are empowered to unearth it. Increased education about doctors’ potential role as gatekeepers into the food aid system may be a short-term solution.^50^ Longer term, gathering data about food insecurity may incentivise the development of effective, evidence-based support structures and equitable, evidence-based policies.

## Conclusions

There is a clear unmet need for further evidence and professional guidance concerning the monitoring and treatment of food insecurity within healthcare settings. Current approaches to food insecurity are inconsistent, both with treatment of other health behaviours such as alcohol and smoking and with recent public statements by professional bodies calling for action to end food poverty. Routine recording of the prevalence and implications of food insecurity within healthcare settings may both allow better care for individual patients and also provide evidence to facilitate effective policy interventions to end hunger, both within and external to the healthcare system. Gathering this data provides a valuable first step in re-framing the social determinants of health as modifiable risks rather than inevitable inequities.

## Data Availability

No data are available.
